# Antimicrobial susceptibility pattern of *Acinetobacter* isolates from patients in Kenyatta National Hospital, Nairobi, Kenya

**DOI:** 10.11604/pamj.2019.33.146.17220

**Published:** 2019-06-26

**Authors:** Victor Moses Musyoki, Moses Muia Masika, Winnie Mutai, Gitau Wilfred, Antony Kuria, Felista Muthini

**Affiliations:** 1 Department of Medical Microbiology, School of Medicine, University of Nairobi, Nairobi, Kenya; 2 Department of Medical Microbiology, Kenyatta National Hospital, Nairobi, Kenya

**Keywords:** *Acinetobacter*, *A. baumannii*, antibiotic resistance, critical care unit

## Abstract

**Introduction:**

Infection due to multidrug-resistant microorganisms is a growing threat in healthcare settings. *Acinetobacter* species specifically *A. baumannii* is increasingly becoming resistant to most antimicrobial agents recommended for treatment. This study aimed to determine the antimicrobial susceptibility pattern of *Acinetobacter* species isolated from patients in Kenyatta National Hospital.

**Methods:**

We conducted a retrospective study based on VITEK 2 (BioMérieux) electronic records capturing identification and antimicrobial susceptibility of *Acinetobacter* isolates from patient samples analyzed between 2013 and 2015 at Kenyatta National Hospital microbiology laboratory. Generated data were analyzed using WHONET and SPSS.

**Results:**

A total of 590 *Acinetobacter* isolates were analyzed. 85% of the isolates tested were multi-drug resistant (MDR). Among the 590 isolates, 273 (46%) were from tracheal aspirates and 285 (48%) from the critical care unit. *A. baumannii* was the most frequently isolated species with high susceptibility to amikacin (77%) and poor susceptibility to ciprofloxacin (69-76%), tobramycin (37%) and meropenem (27%). Both *A. lwoffii* and *A. haemolyticus* had high susceptibility to amikacin (80-100%) and meropenem (75-100%).

**Conclusion:**

*A. baumannii* is resistant to commonly administered antibiotics. There is need for continuous antimicrobial resistance surveillance especially in health care facilities and strengthening of antibiotic stewardship programmes which will contribute to enhancement of infection control policies.

## Introduction

**Figure 1:**
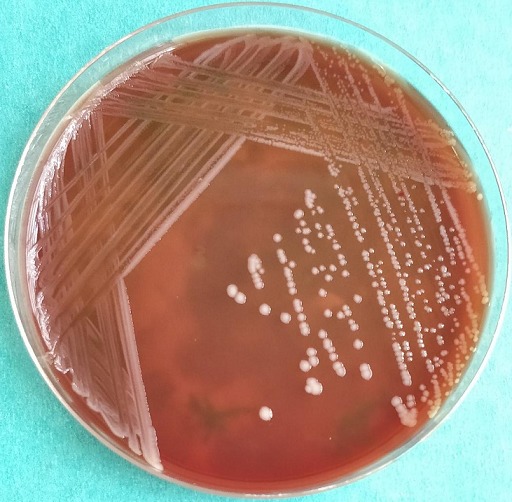
*Acinetobacter* species growing on blood agar

The genus *Acinetobacter* comprises of non-motile gram-negative coccobacilli bacteria. The colonies are 1 to 2mm, non-hemolytic, mucoid, smooth and round on sheep's blood agar after 24 hours of incubation at 37ºC (Figure 1) [1-3]. Most species in this genus have emerged as common pathogens causing community as well as hospital-acquired infections [4, 5]. Hospital-acquired infections are common among patients admitted in the intensive care unit (ICU) and those patients not admitted in the ICU but are immunocompromised. Infections linked to these species include wound infections, urinary tract infections, pneumonia and bacteremia subsequent to trauma, urinary catheters, mechanical ventilators and central venous access catheters respectively. These infections increase the length of hospital stay and risk of hospital death [6]. As a health concern, *Acinetobacter* associated infections are difficult to treat due to the natural tendency of acquisition and spread of multidrug-resistant strains among hospitalized patients and the organisms' different mechanism of antimicrobial resistance [7, 8]. This has contributed to the high morbidity and mortality rate ranging from 27% and 91% especially in immunocompromised patients in the last three decades [9]. Globally, the occurrence of MDR *Acinetobacter*, particularly *A. baumannii* has been reported through several epidemiological studies [10] with a documentation of 10-15% prevalence of *Acinetobacter* resistance to carbapenem, penicillins and fluoroquinolones [11, 12]. *Acinetobacter* species have relatively high resistance to carbapenems, even in countries with high level of awareness and vibrant national nosocomial infection surveillance with an overall low antibiotic resistance [11-13]. However, carbapenems remain the treatment of choice for *Acinetobacter* infections [14]. In two separate studies conducted in Kenya, one study noted that 10% of community-acquired bacteremia was associated with *Acinetobacter* species [13] while in another study that recruited hospitalized patients, *A. baumannii* accounted for 0.9% in wound infections [15]. Management of nosocomial infections remains a challenge in healthcare settings due to the increasing resistance to antimicrobials [16]. Therefore the aim of this study was to evaluate the antimicrobial susceptibility of *Acinetobacter* species isolated from patients in Kenyatta National Hospital (KNH). Previous studies done in Kenya focused on critical care units, we however explored other hospital units.

## Methods

This was a retrospective study. We analyzed electronic laboratory records of *Acinetobacter* isolates from clinical specimens analyzed between 2013 and 2015 at KNH microbiology laboratory. Identification and antimicrobial susceptibility data were retrieved from the VITEK-2 antimicrobial susceptibility system and exported to WHONET through BACLINK. Analysis was done using WHONET version 5.6 and IBM SPSS Statistics version 21. Identification of *Acinetobacter* isolates was done using VITEK-2 Gram Negative identification card (GN83). Clinical specimens were mainly tracheal aspirates, pus, and urine and were analyzed according to the 2015 Clinical and Laboratory Standards Institute guidelines (CLSI M100-S25). The panel of antibiotics tested included amoxicillin/clavulanic acid (20/10 μg), amikacin (30 μg), ampicillin (10 μg), aztreonam (3 μg), ceftazidime (30 μg), ciprofloxacin (5 μg), cefpodoxime (10 μg), ceftriaxone (30 μg), cefotaxime (30 μg), cefuroxime axetil (30 μg), cefuroxime (30 μg), cefazolin (30 μg), cefepime (30 μg), cefoxitin (30 μg), gentamicin (10 μg), meropenem (10 μg), nitrofurantoin (300 μg), norfloxacin (10 μg), piperacillin (100 μg), ampicillin/sulbactam (10/10 μg), trimethoprim/sulfamethoxazole (1.25/23.75 μg), tobramycin (10 μg) and piperacillin/tazobactam (100/10 μg). The laboratory participates in external quality assurance (EQA) coordinated by United Kingdom National External Quality Assurance (UKNEQAS) and World Health Organization-National Institute for Communicable Diseases (WHO-NICD). This helps to evaluate reliability of methods, materials, equipment and staff training impact. The study was approved by the Kenyatta National Hospital-University of Nairobi Ethics and Research Committee.

## Results

**Figure 2:**
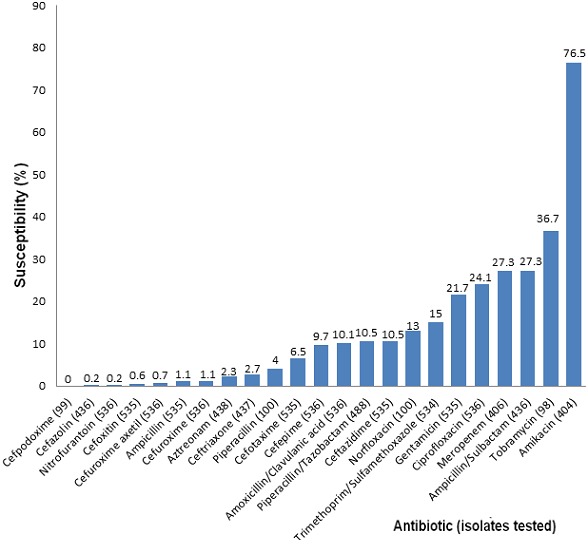
Antimicrobial susceptibility pattern of clinical isolates of *Acinetobacter baumannii*

The study analyzed 590 *Acinetobacter* isolates. Majority of the isolates were from male patients, 380 (52%). The isolates were mainly from tracheal aspirates (273; 46%), pus (130; 22%), urine (93; 16%) and blood (35; 6%). The specimen type for thirty-five (6%) of the isolates was unknown. Other isolates were obtained from peritoneal fluid, pleural fluid, cerebral spinal fluid and tissue. The most frequently isolated *Acinetobacter* species in this study was *Acinetobacter baumannii* (95%); the other isolates included *Acinetobacter lwoffii* (3%) and *Acinetobacter haemolyticus* (1%) (Table 1). Most of the isolates were obtained from samples collected from critical care unit (48%) and internal medicine (13%) with the least obtained from accident and emergency unit (9%) and burns unit (3%). *Acinetobacter baumannii* (n=560) showed high susceptibility to amikacin (77%) and poor susceptibility to tobramycin (37%), meropenem (27%), penicillins (1-27%), fluoroquinolones (13-24%), cephalosporins (0-11%) and trimethoprim-sulfamethoxazole (15%) (Figure 2). Although *Acinetobacter lwoffii* (n=15), *A. haemolyticus* (n=5), *A. junii* (n=1) and other *Acinetobacter* species (n=8) did not meet the CLSI threshold for antibiogram reporting (≥30 isolates each), their AST results were reported due to their microbiological significance. Both *A. lwoffii* and *A. haemolyticus* had high susceptibility to amikacin (80-100%), meropenem (75-100%), ciprofloxacin (80-85%), gentamicin (80-100%) and piperacillin/tazobactam (75-80%); poor susceptibility to aztreonam (23-25%), cefuroxime axetil (20-31%), cefazolin (23-25%) and cefoxitin (20-39%) (Table 2). *Acinetobacter baumannii* isolates from tracheal aspirates, pus, and urine, showed high level of resistance to cephalosporins (65-85%), ciprofloxacin (69-76%) and moderate resistance to meropenem (51-59%) (Table 3). Isolates obtained from blood and other specimens had moderate resistance to cephalosporins (49-63%). *Acinetobacter baumannii* isolates from all specimens showed relatively high sensitivity to amikacin (77-89%). Antibiotic susceptibility varied with hospital units. High resistance to cephalosporins (65-86%) was seen in *A. baumannii* isolates from critical care unit, obstetrics and gynecology, internal medicine ward, accident and emergency, and surgery (Table 4). In summary, *A. baumannii* was the most common species isolated and showed high susceptibility to amikacin (77%), high resistance to cephalosporins (89-100%), fluoroquinolones (76-87%) and meropenem (72%).

**Table 1:** Distribution of *Acinetobacter* isolates by species

Species	*n* (%)
*A*. *baumannii*	560 (95)
*A. lwoffii*	15 (3)
*A. haemolyticus*	5 (1)
*A. calcoaceticus*	1 (0)
*A. junii*	1 (0)
Other *Acinetobacter* species	8 (1)

**Table 2:** Antibiotic susceptibility profile of *Acinetobacter isolates*

Antibiotic	*A. lwoffii*			*A. junii*		*A. haemolyticus*		*Other A. spp*	
	n	% S		n	% S	n	% S	n	% S
Amoxicillin/Clavulanic acid	13		69	1	100	5	100	3	0
Amikacin	13		100	1	100	5	80	2	100
Ampicillin	13		46	1	0	5	80	3	0
Aztreonam	13		23	0	0	4	25	0	0
Ceftazidime	13		62	1	0	5	60	3	0
Ciprofloxacin	13		85	1	100	5	80	3	33
Cefpodoxime	0		0	1	0	1	0	3	0
Ceftriaxone	13		62	0	0	4	75	0	0
Cefotaxime	13		77	1	0	5	80	3	0
Cefuroxime axetil	13		39	1	0	5	20	3	0
Cefuroxime	13		39	1	0	5	80	3	0
Cefazolin	13		23	0	0	4	25	0	0
Cefepime	14		100	1	100	5	80	3	0
Cefoxitin	13		39	1	100	5	20	3	0
Gentamicin	14		100	1	100	5	80	3	33
Meropenem	10		100	0	0	4	75	0	0
Nitrofurantoin	13		31	1	0	5	20	3	33
Norfloxacin	1		0	1	100	1	100	3	33
Piperacillin	12		0	1	100	1	100	3	0
Ampicillin/Sulbactam	13		85	0	0	4	75	0	0
Trimethoprim/Sulfamethoxazole	13		85	1	0	5	60	3	0
Tobramycin	3		33	1	100	1	0	1	0
Piperacillin/Tazobactam	12		75	1	100	5	80	5	20

Key: n= number of isolates, % S = percentage susceptible

**Table 3:** Antibiotic resistance of *Acinetobacter baumannii* isolates by specimen type

Antibiotic	Tracheal aspirate (n = 266)	Pus (n =127 )	Urine (n = 85)	Blood (n = 31)	Others (n = 22)	N/I (n = 30)
Ceftazidime	84	85	83	54	63	80
Ceftriaxone	73	73	65	51	54	0
Cefotaxime	84	85	82	51	63	80
Cefepime	82	72	77	49	58	83
Ciprofloxacin	71	69	76	43	46	74
Meropenem	59	49	51	37	38	0
Amikacin	17	13	23	11	13	0

KEY: N/I; Not Indicated; % = percentage. Others included – peritoneal fluid, pleural fluid, cerebral spinal fluid, high vaginal swabs, and tissue

**Table 4:** antibiotic resistance of *Acinetobacter baumannii* isolates by Hospital unit

Antibiotic	Critical Care Unit (n =285)	Internal medicine (n = 76)	Accident & Emergency (n = 54)	Surgery (n = 45)	Pediatrics (n = 32)	Obs & Gyn (n = 24)	Burn unit (n = 17)	Not indicated (n = 36)	Others (n =21)
Ceftazidime	86	75	82	84	50	75	77	81	76
Ceftriaxone	73	65	78	69	41	67	53	8	76
Cefotaxime	87	74	83	84	47	75	77	80	76
Cefepime	86	67	76	71	40	75	35	83	71
Ciprofloxacin	76	68	70	56	38	58	59	69	71
Meropenem	61	47	52	51	22	46	18	6	48
Amikacin	20	12	17	11	6	13	12	0	19

% = percentage; Others include surgical and medical outpatient clinic

## Discussion

In this study, we recorded 95% of *Acinetobacter baumannii* from all the samples analyzed. Other species were detected in low numbers and included *Acinetobacter lwoffii* (3%), *A. haemolyticus* (1%), *A. calcoaceticus* (n=1) and *A. junii* (n=1). A similar observation was noted in similar studies in India [17, 18] and Nigeria [19] where *A. baumannii* and *A. lwoffii* were the predominant species. The predominance of *Acinetobacter baumannii* is most likely due to its ability to survive for a long period in the hospital environment, the potential to respond to selective environment pressure and its non-fastidious nature. Based on hospital units and specimen type, majority of the isolates were obtained from samples collected from the critical care unit (48%) and tracheal aspirates (46%) respectively. Consequently, high level of resistance (71-86%) to the commonly used antibiotics was noted in isolates from these units. These findings concur with results from similar studies conducted in India [18], Iran [20] and Sudan [21]. Several factors including immunosuppressed hosts, previous use of antibiotics, patients with severe underlying diseases, duration of hospital stay, the increasingly invasive diagnostic procedures and more frequent use of antibiotics in ICU have been shown to contribute to the occurrence of *Acinetobacter* and especially *A. baumannii* [1, 22]. The highest number of isolates were recovered from tracheal aspirates, pus and urine samples, consistent with results from studies in several hospitals in India [17, 22] and Morocco [9]. These results were in contrast with those reported in two separate studies done in India [23, 24] which showed highest number of isolates were from tracheal aspirate followed by blood and pus respectively. The rate of isolation from different clinical samples is mostly influenced by the differences in the sample types, antibiotic usage, previous history of specific site colonization, infection control practices and a times the type of health facility.

Generally, *A. baumannii* exhibit resistance to multiple antibiotics. Third and fourth generation cephalosporins, carbapenems and fluoroquinolones are the most commonly used antibiotics in treating infections in hospitalized patients. In our study, a total of 502 (85%) *Acinetobacter* isolates, mostly *A. baumannii* were multidrug resistant. Multi-drug resistant (MDR) was defined as non-susceptibility to at least one agent in three or more antimicrobial classes. The high proportion of multidrug resistant *A. baumannii* have been reported in other studies globally with major impact reported in Asian countries including Malaysia, India, and Pakistan [25-27]. *A. baumannii* isolated showed high resistance to majority of the antibacterial agents tested and high susceptibility to amikacin (77%). Our findings on resistance to the commonly prescribed cephalosporins are consistent with results from several previous studies in other parts of the world showing high resistance to third and fourth generation cephalosporins, for instance a survey in tertiary hospitals in Colombia, Turkey, Romania, and Sudan reported resistance to cefotaxime, ceftazidime, and cefepime with proportions ranging from 84% to 98% [21, 22, 27, 28]. In contrast, similar studies in the Netherlands and India reported resistance rate of 16%-56% to ceftazidime and cefepime [29, 30]. The high resistance to Beta-lactam antibiotics in *Acinetobacter* species especially *A. baumannii* is most likely associated with the production of B-lactamases including *TEM-1*, *TEM-2* and CARB-5, *AR-1*, *ACE-1, 2, 3, 4* and the ESBL whose genes are either chromosomally or plasmid located. This may lead to alteration of penicillin-binding proteins and reduction in permeability to antimicrobials conferring some inherent resistance [1, 31]. On fluoroquinolones, we observed a high level of ciprofloxacin resistance (69-76%) in *A. baumannii* similar to findings of studies done in different referral hospitals in Turkey (90%), India (86%), Iran (80-82%) and Sudan (91%) [16, 21, 24, 32, 33]. This observation is contrary to what was initially reported in the same Hospital (KNH) in 2009 where they reported up to 100% susceptibility to ciprofloxacin [15]. The *A. baumannii* resistance to fluoroquinolones could be attributed to the structural modifications of the DNA gyrase subunits by *gyrA* and *parC* gene mutations. Additional explanation to this is the decreased uptake of the antimicrobials due to altered outer membrane (protein) leading to the early development of *gyrA* and *parC* resistance genes [1, 34] and the efflux systems that decrease intracellular drug accumulation [35].

Carbapenems for a long time have been the most potent drugs in the treatment of *A. baumannii* infection. In several parts of sub-Sahara Africa and other parts of the world, *A. baumannii* has been reported to exhibit resistance to carbapenems which is not the case with other *Acinetobacter* species. In our study, *A. baumannii* was resistant to meropenem (72%), compared to *A. lwoffii* and *A. haemolyticus* which recorded a high susceptibility of 100% and 75% respectively. Studies in other countries have however reported a slightly higher rate of resistance to meropenem (80-87%) in *A. baumannii* [28, 35, 36]. These findings are in contrast with several reports from previous studies where *A. baumannii* resistance to meropenem was remarkably lower (52-62%) than what we observed [17, 19, 37]. The high resistance to carbapenems and especially meropenem is attributed to prolonged empirical treatment duration with the drug. Further, resistance is also driven by production of carbapenemase enzymes categorically class B Metallo-beta-Lactamases (MBLs) and class D oxacillinases. Additional mechanisms involve efflux pump and impermeability associated mutations altering the porins expression [9, 28, 38]. Although aminoglycosides like amikacin and tobramycin retain activity against *Acinetobacter* species and especially *A. baumannii*, resistance to these drugs is emerging as demonstrated in this study where 23% of *A. baumannii* isolates exhibited resistance to amikacin. Similar findings have been reported in India [15] and Iran [31]. Previous studies have noted moderate (55%) to high resistance (78%) to amikacin [25, 35, 37, 38-40]. This study therefore highlights the occurrence of antibiotic resistant *Acinetobacter* species in a hospital setup considerably *A. baumannii* which showed high resistance to first line treatment regime of *Acinetobacter* associated infections. The major limitation in this study was the missing socio-demographics, clinical, and previous antibiotic use information which strongly underpins the importance of an integrated laboratory information management system in data capture. Another limitation is the fact that we were not able to confirm if all *Acinetobacter* isolates were causing infection or were just colonizers. This requires the correlation of laboratory results with the clinical presentation of the patient.

## Conclusion

We report a high proportion of *Acinetobacter* isolates from samples obtained from critical care unit (48%) and tracheal aspirates (46%). Besides, *A. baumannii* was the most common species isolated and it demonstrated high susceptibility to amikacin (77%) and high resistance to commonly administered antibiotics such as cephalosporins, fluoroquinolones, penicillins, and meropenem. With the emergence and increase of MDR *Acinetobacter*, this study provides further evidence of the need for continuous surveillance of *A. baumannii* resistance patterns and enforcement of antibiotic stewardship programs in healthcare settings. There is need for further research on molecular mechanisms of resistance to monitor the epidemiology of MDR *A. baumannii* and combat antimicrobial resistance.

### What is known about this topic

- *Acinetobacter* is a non-motile, gram-negative coccobacillus that is found in the environment and colonizes the human body;
- *Acinetobacter* is a nosocomial pathogen associated with high mortality and morbidity especially among the immunocompromised patients;
- It's known for its intrinsic antibiotic resistance mechanism and the ability to rapidly acquire resistance genes.

### What this study adds

- *Acinetobacter baumannii* was the most frequently isolated species and demonstrated high susceptibility to amikacin;
- Other species isolated were *A. lwoffii* and *A. haemolyticus*. Both had high susceptibility to amikacin, meropenem, ciprofloxacin and gentamicin, but showed poor susceptibility to cephalosporins;
- Critical care units and tracheal aspirate had the highest proportion of *A. baumannii* isolates and recorded high resistance to commonly used antibiotics such as penicillins, fluoroquinolones, cephalosporins and meropenem.

## Competing interests

The authors declare no competing interests.
